# Application Effect of Transparent Supervision Based on Informatization in Prevention and Control of Carbapenem-Resistant *Klebsiella pneumoniae* Nosocomial Infection

**DOI:** 10.1155/2022/2193430

**Published:** 2022-10-25

**Authors:** Xiaoqin Wu, Quansheng Lu, Guo Feng, Hongxia Kan, Liran Shi

**Affiliations:** ^1^The People's Hospital of Jiawang of Xuzhou, Xuzhou 221011, China; ^2^Xuzhou Medical University, Xuzhou, China

## Abstract

**Objective:**

To explore the effect of transparent supervision model on the prevention and control of carbapenem-resistant *Klebsiella pneumoniae* (CRKP) nosocomial infection and the value of the autoregressive integrated moving average (ARIMA) model in predicting the incidence of CRKP infection.

**Methods:**

A total of 46,873 inpatients from Jiawang District People's Hospital of Xuzhou between January 2019 and December 2019 (prior to COVID-19 prevention and control) were selected as the preintervention group and 45,217 inpatients from January 2020 to December 2020 (after the COVID-19 prevention and control) as the postintervention group. We performed transparent supervision on CRKP patients detected by the real-time monitoring system for nosocomial infection. Incidence and detection rate of CRKP, utilization rate of special grade hydrocarbon enzyme alkene antibiotics, hand hygiene compliance rate, qualified rate of ATP tests on surface of environmental objects, and execution rate of CRKP core prevention and control were compared between the two groups.

**Results:**

Transparent supervision of CRKP-infected patients was conducted daily from January to December 2020, which resulted in the following: (a) the infection rate of CRKP decreased in a fluctuating manner, and the actual value of hydrocarbon alkene use rate was basically the same as the predicted value with an overall decreasing trend; (b) after the intervention, hand hygiene compliance rate increased from 53.30% to 70.24% (*P* < 0.001) and the ATP qualified rate increased from 53.77% to 92.24% (*P* < 0.001); (c) the fitted value of the ARIMA model was in good agreement with the actual value. The incidence of CRKP infection and the utilization rate of carbene antibiotics were also in good agreement with the predicted value. The average relative errors were 11% and 10.78%.

**Conclusions:**

During the COVID-19 outbreak in 2020, the ARIMA model effectively fit and predicted the CRKP infection rate, thereby providing scientific guidance for the prevention and control of CRKP infection. In addition, the transparent supervision intervention model improved the hand hygiene compliance and environmental hygiene qualification rates of medical staff, effectively reducing CRKP cross-infection in the hospital.

## 1. Introduction

With the widespread use of carbapenems in recent years, the rapid growth of CRKP has caused great attention worldwide. According to the China Antimicrobial Resistance Surveillance System (CARSS) in 2019, detection rate of CRKP in China increased from 6.4% in 2014 to 10.9% in 2019, with the highest rate 32.8% in Henan Province, which was just 9.4% in 2014. The strong drug resistance of CRKP to almost all carbapenem antibiotics results in the unsatisfied effect of anti-infective therapies [1, 2]. CRKP not only affects the prognosis of patients, but also increases hospitalization costs and mortality risk [3]. A study by Falagas et al. [4] showed that the mortality rate of CRKP patients was as high as 14.6%, and once CRKP infection occurred, the hospitalization costs increased significantly. According to another study by Huang et al. [5], the average hospitalization cost of patients with CRKP infection was approximately 23,000 US dollars, significantly higher than that of patients with carbapenem-susceptible *Klebsiella pneumoniae* was about 18,000 US dollars, and multiple drug-resistant organisms (MDRO) frequently resulted in hospital infection outbreaks. Ninety-eight cases have been attributed to carbapenemase-producing *Enterobacteriaceae*, according to the literature [6, 7]. Studies have reported MDRO outbreaks due to poor compliance to hand hygiene, personal protective equipment (PPE) shortages, and high antibiotic use during the COVID-19 pandemic [8]. Another study by Sun Jin L. and Fisher D. [9] stated that due to the lack of MDRO infection control measures and the irrational use of antibacterial drugs, MDRO infection outbreaks have also occurred during COVID-19. A study showed that during the COVID-19 epidemic from 12 November to 19 December 2020, a CRKP outbreak occurred in the ICU of a tertiary hospital, and the risk factors of CRKP outbreak were analyzed related to invasive procedures [10]; the drug resistance and virulence analysis of an outbreak of KPC *Klebsiella pneumoniae* ST15 strain in a tertiary hospital in China found that there was high drug resistance and CRKP should be strictly monitored [11]. CRKP infection outbreak necessitates a large number of medical and social resources [12]. The CRKP outbreaks increase requirements and present new challenges for preventing and controlling nosocomial infections [13]. In 2017, in response to MDRO and CRKP outbreaks, the World Health Organization (WHO) emphasized following the guidelines for the prevention and control of carbapenem-resistant *Enterobacteriaceae*, *Pseudomonas aeruginosa*, and *Acinetobacter baumannii* in medical institutions [14]. Therefore, in order to strengthen the implementation of core measures for CRKP infection prevention and control, improve CRKP infection prevention and control capabilities and compliance with core prevention and control measures, optimize CRKP infection prevention measures and rationally use carbapenems. Since 2020, the COVID-19 epidemic has entered the stage of normalized prevention and control, under the premise of doing a good job in epidemic prevention and control, it has carried out information-based methods to transparently supervise the core measures of CRKP infection prevention and control and use information-based platforms to promote the rational use of special-grade antibiotics. Process control has achieved good results, the report is as follows.

## 2. Materials and Methods

### 2.1. Materials

Microbiology tests were performed from January to December 2020, all departments and inpatients with positive CRKP were included in this study. With the aid of informatization, the report was electronically sent in the form of critical values. The mobile application management system was used to implement precise prevention and control of CRKP and to transparently supervise compliance of CRKP prevention and control measures, use of carbapenems and antibiotics, and data disclosure. Moreover, the control group was comprised of the number of hospitalized patients and the number of CRKP strains examined from January to December 2019. This study was approved by the ethics committee of The Hospital of Xuzhou Medical University Jiawang branch of Xuzhou (2019 (010)).

### 2.2. Methods

#### 2.2.1. Transparent Supervision on the Use of Carbapenems Based on Informatization

MDRO early warning platform established by the hospital infection real-time monitoring system was used to carry out MDRO network automatic early warning and reminder, and the hospital infection professionals utilized the mobile application terminal to interact with clinicians and bed nurses. The antibacterial drug use management system was launched for the carbapenem drug project management information platform to implement the carbapenem antibacterial drug project management. The application management background implemented the CRKP infection prevention and control process and the use of carbapenem antibacterial drugs. The case tracking method and plan-do-check-action (PDCA) cycle continuously improved the prevention and control of CRKP infection and conducted transparent supervision of the process, results, and use of carbapenems in the prevention and control of CRKP infection.

#### 2.2.2. Transparent Supervision of Process Indicators Such as Hand Hygiene and Contact Isolation

A transparent supervision team was established led by the deputy dean in charge, and members of the medical department, infection management department, microbiology room, nursing department, pharmacy department, and the hospital quality control team of the department formed a transparent supervision team to transparently supervise the CRKP prevention and control process, that is, (1) daily infection management for CRKP patients alerted by the hospital infection real-time monitoring system, full-time staff will go to the bedside to supervise the timeliness of the implementation of contact isolation prevention and control measures; (2) strengthen hand hygiene compliance, 5 moments of hand hygiene and the implementation of the six-step handwashing method; (3) use ATP fluorescence detector to sample the cleanliness of the surfaces of the environmental objects in the cleaning unit around CRKP patients to judge whether the cleaning is qualified or not. Strengthen the supervision of use, and strictly implement the approval system for the use of antibacterial drugs at special levels. Every day from the real-time monitoring system of nosocomial infection to monitor the inspection situation of therapeutic antibiotics and provide timely feedback.

#### 2.2.3. Implementation of Informatization Control for Carbapenems Was Based on the Existing Antibiotics Management System in the Hospital

The contents were as follows: (a) limited the permission of carbapenem prescriptions, which could be opened by director of the department, associate chief and chief physicians; (b) associate chief and chief physicians of respiratory and critical care, infectious disease department and intensive care unit could directly prescribe carbapenems without consultation authorization; (c) if carbapenems were needed in other departments, they must be consulted by the expert group before prescribed by authorized physicians; (d) when giving medical orders, doctors were required to choose whether consultation and microbial samples delivery are needed prior to the dialog box of further medical advice; (e) clinical pharmacist review system was added to the hospital information system (HIS), on which carbapenems orders were reviewed and verified by clinical pharmacists online before prescriptions, thus to achieve transparent supervision on the process of carbapenem utilization; (f) monthly statistics on rational use of carbapenems were reported at the department director regular meeting so as to realize transparent supervision on the results of carbapenem utilization.

#### 2.2.4. Disclosure of Outcome Index and Achievement of Continuous Improvement

Attention was fixed on CRKP with continuous increasing detection rate in recent years, according to the real-time monitoring data of nosocomial infection. The transparent supervision group created a WeChat group and timely posted prevention and control result indicators of each department regarding isolation prescriptions, usage and preuse testing rate of therapeutic antibiotics, hand hygiene compliance, and qualified rate of environmental hygiene cleaning and disinfection, as well as gave feedback on problems by mobile phone application.

### 2.3. ARIMA Modeling

First, the time series data of CRKP monthly infection rate was selected, and the time series diagram was used to test the stationarity. It can be seen from the time series diagram that the sequence exhibited a certain fluctuation. To further test the stationarity of the series, augmented Dickey–Fuller test (ADF) was used to investigate. The *t* value was −3.40, *P*=0.02, the series was a nonstationary time series, and the difference method was used for stationarity processing. ADF test statistic *t* = −5.07, *P*=0.0006, there was no unit after the first-order difference. The root phenomenon is a first-order single integral sequence, and a time series ARIMA mixed model should be established.

### 2.4. Order Identification of the Model

The autocorrelation function (ACF) and partial autocorrelation function (PACF) diagrams of the stationary time series were used to determine the order of the model preliminarily, it was found that the order of the ACF and PACF diagrams of the first-order difference was not obvious, while the second-order difference sequence is a stationary sequence. In order to determine the optimal model, the minimum criteria of the Schwarz criterion (SC) and Akaike information criterion (AIC) were used to judge the optimal order of the model. The relevant test results demonstrated that the SC of ARIMA (3, 2, and 1) was 2.349, and the AIC value was 2.548, which was the smallest among the three models, so ARIMA (3, 2, and 1) was finally selected for the establishment of this model.

### 2.5. Model Building and Testing

The ARIMA (3, 2, and 1) model was run through statistical software to test the stability of the model further, and autocorrelation and heteroscedasticity tests were performed on the residual sequence of the model. We performed parameter estimation to test whether it was statistically significant, and hypothesis testing was performed to diagnose whether the residual sequence was white noise. If all were satisfied, the model construction was deemed reasonable. If none of them were satisfied, the model had to be rebuilt.

### 2.6. Model Predictions

The model was used to predict the original data from January to December 2020 and then predicted, and the original value was used to calculate the relative error to obtain the future infection incidence of the original sequence to determine the optimal model.

### 2.7. Statistical Analysis

SPSS 19.0 statistical analysis software was used to model the ARIMA, and the parameters of the selected model were estimated and tested. The test level *α* = 0.05 and *P* < 0.05was considered statistically significant.

## 3. Results

### 3.1. Time Series and Stationarity Test of CRKP Infection Rate

**Figure 1:**
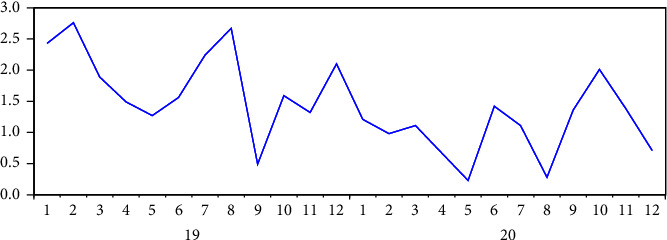
Time series diagram of CRKP infection rate from January 2019 to December 2020.

The CRKP nosocomial infection rate from January to December 2019 and January to December 2020 and the comparison of the CRKP infection rate before and after the implementation of transparent supervision (January to December 2019) were used for time series analysis; the time series graph showed that the monthly infection rate exhibited fluctuating state, and the incidence peak period occurred every year. After the series was processed for stationarity by the first-order difference method, the ADF test statistic *t* value was −5.071 and the probability was 0.0006. Compared with the 1% level critical value, it was less than the horizontal critical value; the CRKP infection rate series after the final treatment was a first-order difference from a stationary series. The results are shown in Figure 1 and Table 1.

### 3.2. Model Fitting

**Figure 2:**
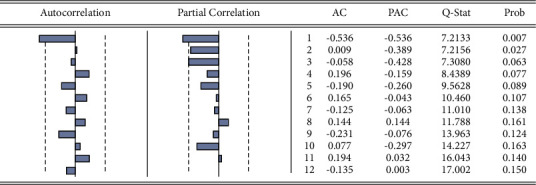
Series diagram of model residual correlation function.

The ACF and PACF plots of the time series after stationary were used to determine the order of the model preliminarily. The third-order trailing is shown in Figure 2, *p* was taken as 3, 2, and 1, and the value of *q* was taken as 1. The SC and AIC minimum criteria were used for modeling, and the ARIMA model was run through statistical software for parameter estimation and hypothesis testing. The result regression table *R*-squared was 0.78, the model fit was good, and the adjusted coefficient of determination adjusted *R*-squared was 0.74; the model was well established, as shown in Table 2.

### 3.3. Model Prediction

**Figure 3:**
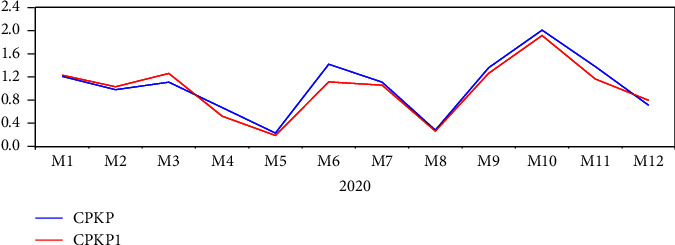
Series diagram of CRKP infection rate in 2020.

We used ARIMA (3, 2, and 1) for predictive analysis and to draw a sequence diagram between the predicted sequence and the original sequence, where CPKP was the original sequence, CPKP1 was the predicted sequence, and the predicted value was consistent with the actual value sequence diagram, the obtained results are shown in Figure 3. The constructed model was used to predict the original data from January to December 2020; the relative error of prediction in most periods was less than 15%. The maximum relative error of prediction was April 2020, while the relative error was 22.09%. The minimum was January 2020, while the relative error value was 1.71%, and the average relative error was 11%. The fitting prediction results are shown in Table 3.

### 3.4. Actual Value and Predicted Value of Carbapenem Use Rate in 2020

The model was used to predict the original data from January to December 2020, and the relative error between the predicted value and the original value was calculated. From Table 4, it can be seen that the relative error of prediction in most periods was less than 15% with an average of 10.78%, indicating a good prediction effect. The maximum relative error of prediction was February 2020 with a relative error 17.84%, while the minimum was April 2020 with a relative error 3.45%. The time series diagram of the predicted value and the actual value was basically consistent, as shown in Table 4.

### 3.5. Hand Hygiene of Medical Staff

Before and after the intervention, the hand hygiene compliance rate of medical staff (70.24% vs. 53.3%) and the hygiene accuracy rate (98.03% vs. 89.72%) were statistically significant (*P* < 0.001). Glove wearing rate in nonpractice hand hygiene before and after intervention (70.11% vs. 61.84%) was statistically significant (*P* < 0.05), the results are shown in Table 5.

### 3.6. Environmental Hygiene Monitoring in the Two Groups before and after the COVID-19

The implementation of core prevention and control measures such as disinfection of environmental surfaces, disposal of medical waste, and dedicated personnel for diagnosis and treatment equipment before and after the intervention, there was a statistically significant difference in the monitoring results of environmental hygiene between the two groups (*P* < 0.05) (Table 6).

## 4. Discussion

1. Time series analysis has been widely used as a prediction for the epidemic of infectious diseases in the field of public health and is a classical statistical method for analyzing and predicting the change trend of variables or outcomes [15, 16]. Balinskaite et al. [17] used intermittent time series regression to conclude that the implementation of national financial incentive policy could reduce the use of antibiotics and improve the quality of antibiotics prescriptions. This study initially conducted a time series analysis through the modeling of CRKP monthly infection rate to predict the change in the trend of CRKP infection rate. The results showed that the predicted value of the CRKP monthly infection rate was almost the same as the actual value in most months (relative error of less than 15%) and average relative error 11%, which indicated that the CRKP monthly infection rate time series analysis mode had a good applicable effect on early warning of nosocomial infection. It is fully explained that the predicted value is able to provide a scientific basis for early prevention and control of CRKP infection epidemic. However, in April and June 2020, the error between the actual and predicted values was relatively large (relative errors were 22.09% and 21.55%). The actual and predicted values of the CRKP infection rate from January to December 2020 showed a slight fluctuation and a downward trend. This may be due to the initial stage of the COVID-19 outbreak in January 2020 to the normalized epidemic prevention and control period after April. Due to the strict prevention and control of the COVID-19 epidemic, the infection control work has received unprecedented attention, and the implementation of various infection control measures, such as hand hygiene, has been emphasized [13]. This has resulted in the further control of the incidence of nosocomial infections, demonstrating the positive role of epidemic prevention and control in reducing nosocomial infections.
2. In this study, the real-time monitoring data of nosocomial infection from 2019 to 2020 was selected. Before the implementation of transparent supervision, the detection rate of CRKP was 29.42%, which was significantly higher than 11.60% among third-class hospitals in 2019 according to CARSS, thus it is necessary to carry out process control for nosocomial infection of CRKP. From the time series analysis, it could be seen that the actual value and predictive value of the special grade carbapenems use rate showed a fluctuating downward trend from January to December 2020, which might benefit from (1) the transparent supervision on various core prevention and control measures of CRKP; (2) the reinforcement of the rational and standardized use of special grade carbapenems; (3) the improvement of the submission and rational use of microorganisms before the use of therapeutic and special grade antibiotics; (4) the enhancement of the quality of nosocomial infection and the effective implementation of core prevention and control measures of CRKP.
3. Implementation of evidence-based hand and environmental hygiene is crucial in CRKP prevention and control [18]. Another meta-analysis [19] has also proved that hand hygiene, environmental cleaning, purpose-specific devices, and limited antibiotics could inhibit the spread of carbapenem-resistant *Enterobacteriaceae* (CRE) and CRKP. Our study seeks to understand and explain the role of hand hygiene and comes to similar conclusions. Hand hygiene compliance and ATP qualified rate have statistical improvements after the intervention of transparent supervision on five moments and six-step hand washing methods among medical staffs and CRKP patients (*P* < 0.05). The importance of hand hygiene is evident in the prevention and control of CRKP, especially the medical staff who have experienced the COVID-19 epidemic has significantly improved their awareness and implemented hand hygiene, which was brought by the psychological pressure brought by the spread of the epidemic. This study also showed that the lower rate of hand hygiene compliance while wearing gloves was related to the increased use during the epidemic. Although hand hygiene compliance improved during COVID-19, the occurrence of COVID-19 did not promote medical workers. However, with proper glove use, there may also be unnecessary overuse. Therefore, education and training of medical staff should be strengthened to encourage hand hygiene regardless of whether they need to wear gloves under the guidelines for hand hygiene in medical institutions issued by WHO in 2009 [20]. After taking off gloves, meticulous hand hygiene should be practiced.
4. Reasons for cross-spreading and drug resistance of CRKP, improper infection prevention and control measures lead to cross-infection of CRKP in hospitals, and the unreasonable and extensive use of antibiotics makes it highly resistant [19]. CRKP can contaminate the diagnosis and treatment environment, and such equipment can lead to the accumulation or outbreak of nosocomial infections. Due to multidrug resistance, strong pathogenicity, and rapid spread, CRPK could be very harmful [21]. Some studies [22, 23] found that in the International Component for Unicode (ICU) nosocomial infection outbreak, 22.88% of the source of infection came from the environment and 15.09% was from contaminated medical equipment. Thorough cleaning and disinfection of the environment and surfaces can remove many pathogenic microorganisms mixed with dirt and effectively reduce the spread of bacteria. The findings of this study showed that after the transparent supervision of the cleaning and disinfection of the surface of environmental objects, the qualified rate of cleaning and disinfection of the environment and object surfaces was statistically significant (*P* < 0.05). During the global outbreak of COVID-19 in 2020, the common points of COVID-19 epidemic prevention and control were standard precautions, environmental cleaning, disinfection, and isolation, similar to the MDRO prevention and control. The COVID-19 epidemic prevention and control promoted environmental cleaning and the implementation of disinfection and isolation measures which cut off the transmission route of CRKP, resulting in a decrease in the CRKP infection rate.
5. The prudent use of carbapenems is an essential measure for preventing CRKP infection in patients. The results of this study showed that an information-based carbapenem platform should be established to implement transparent supervision of the use of carbapenems and to regulate the authority of clinicians to use carbapenems. From January to December 2020, it can be seen that the actual and predicted values of carbapenem usage rates were relatively high in most periods. The error was less than 15%, indicating that the prediction effect was good, the average relative error was 10.78%, and the time series diagram between the predicted value and the actual value was consistent. After the intervention, the utilization rate of carbapenems was significantly lower than before. The reason for the decrease was the online real-time review by clinical pharmacists, which controlled the abuse of carbapenems by clinicians and reduced the number of carbapenems. Studies have shown that CRKP-infected patients are resistant to almost all *β*-lactam antibiotics, including carbapenems, cephalosporins, and aztreonam, and combined clinical treatment results in a substantial increase in hospitalization costs. In addition, exposure to antibiotics will increase the risk of CRKP infection [24, 25]. To reduce drug resistance, the rational use of antibiotics should be strengthened.

At this stage, various threats such as the COVID-19 epidemic, infection outbreaks, and multidrug-resistant bacteria have put higher requirements for infection control work. The application of the transparent supervision management model to practice CRKP prevention and control and the rational use of antibacterial drugs has reduced the infection rate of CRKP, which scientifically confirmed the effectiveness of the transparent supervision model. At present, the transparency and disclosure of relevant indicators for infection management in China is still in the exploratory stage, and we can attempt to learn from the monitoring of multidrug-resistant bacteria conducted in the United Kingdom and Sweden [26, 27]. The transparent supervision and intervention mode of information technology has improved the compliance of medical staff with core prevention and control measures such as hand hygiene, reduced the use rate of carbapenems, and effectively reduced the cross-transmission of CRKP and the generation of drug resistance. This reduces the incidence of CRKP infection; the ARIMA model can effectively fit and predict the CRKP infection rate and provide scientific guidance for the prevention and control of CRKP infection, which is of great significance to ensuring medical quality and patient safety.

## Figures and Tables

**Table 1:** First order differential ADF of CRKP infection rate.

		*t*-statistics	Prob.^*∗*^
Augmented Dickey–Fuller test statistics		−5.071	0.0006
Test critical values	1% level	−3.788	
	5% level	−3.012	
	10% level	−2.646	

**Table 2:** Model parameters estimation.

Variables	Coefficients	Std. errors	*t*-statistics	Prob
AR(3)	−0.453	0.172	−2.630	0.018
AR(2)	−0.709	0.228	−3.102	0.007
AR(1)	−0.644	0.251	−2.566	0.022
MA(1)	−0.998	0.265	−3.760	0.001
*R*-squared	0.784	Mean dependent		−0.023
Adjusted *R*-squared	0.740	S.D. dependent		1.401
S.E. of regression	0.714	Akaike info criterion		2.349
Sum-squared residual	7.649	Schwarz criterion		2.547
Log likelihood	−18.316	Hannan–Quinn criterion		2.382
Durbin–Watson statistics	2.276			

**Table 3:** Actual and predicted value of CRKP infection rate in 2020.

Months	Actual values	Predictive values	Relative errors (%)
	Infection rate (%)	Infection rate (%)	
2020-01	1.21	1.23	1.71
2020-02	0.98	1.03	5.11
2020-03	1.11	1.26	13.58
2020-04	0.67	0.52	22.09
2020-05	0.23	0.18	18.03
2020-06	1.42	1.11	21.55
2020-07	1.11	1.06	4.74
2020-08	0.28	0.26	5.86
2020-09	1.36	1.26	7.02
2020-10	2.01	1.91	4.66
2020-11	1.38	1.16	15.54
2020-12	0.71	0.79	12.11

*Note.* Relative error = │(actual value − predicted value)/actual value│*∗*100%.

**Table 4:** Actual and predicted value of carbapenem use rate in 2020.

Months	Utilization actual	Usage forecast	Relative errors (%)
2020-01	2.07	1.93	6.79
2020-02	2.48	2.04	17.84
2020-03	2.18	1.98	9.17
2020-04	1.81	1.87	3.45
2020-05	1.99	1.75	12.09
2020-06	1.94	1.69	13.01
2020-07	2.46	2.21	9.99
2020-08	2.42	2.21	8.71
2020-09	2.3	2.07	9.88
2020-10	1.96	1.68	14.33
2020-11	2.07	1.90	8.38
2020-12	1.73	1.46	15.73

*Note.* Relative error = │(actual value − predicted value)/actual value│*∗*100%.

**Table 5:** Comparison of hand hygiene before and after COVID-19.

Hand hygiene indications	Preintervention 2019			Postintervention 2020			*χ * ^2^	*P*
	Number of investigation cases	Number of qualified cases	Pass rate (%)	Number of investigation cases	Number of qualified cases	Pass rate (%)		
Hand hygiene compliance rate	968	516	53.30	1089	765	70.24	62.613	≤0.001
Hand hygiene accuracy	516	463	89.72	765	750	98.03	42.340	≤0.001
Wear gloves instead of hand hygiene*∗*	425	298	70.11	380	235	61.84	6.141	0.013
Hand sanitizer consumption qualification rate*∗*	26	15	57.69	25	20	80.00	2.946	0.086
Hand sanitizer consumption is acceptable*∗*	26	13	50.00	25	22	88.00	8.548	0.003

*Note.* Qualification rate requirements for wearing gloves instead of hand hygiene are as follows: if hand hygiene is not performed but wearing gloves instead of hand hygiene is marked as qualified, the qualified requirements for the consumption of hand sanitizer in the department is ≥10 ml/bed*∗*day; the consumption of hand sanitizer in the department of eligibility requirements is ICU ≥ 30 ml/bed*∗*day and general ward ≥ 5 ml/bed*∗*day.

**Table 6:** Implementation of cleaning and disinfection of the surrounding measures for CRKP patients before and after COVID-19.

Items	Preintervention 2019			Postintervention 2020			*χ * ^2^	*P*
	Number of investigation cases	Number of qualified cases	Pass rates^*∗*^(%)	Number of investigation cases	Number of qualified cases	Pass rates^*∗*^(%)		
Ventilator and monitor panel	44	21	47.73	36	34	94.44	20.114	0.001
Bedside table and bed rail	39	17	43.59	33	30	90.91	17.658	0.001
Treatment car and nursing car	31	16	51.61	26	23	88.46	8.886	0.003
Medical related items	38	21	53.57	23	20	86.96	6.537	0.011
Infusion or syringe pump surfaces	26	17	65.38	21	20	95.24	6.181	0.013
Shaker handle (remote control)	58	32	55.17	39	35	89.74	13.047	≤0.001
Hand sanitizer button	85	65	76.47	46	42	91.31	5.745	0.017

*Note.* Use an ATP fluorescence detector to monitor cleanliness of the environment and object surface, and RLU ≤ 100 is qualified.

## Data Availability

All data generated or analyzed during this study are included within this article.

## References

[1] Hammoudi Halat D., Ayoub Moubareck C. (2020). The current burden of carbapenemases: review of significant properties and dissemination among gram-negative bacteria. *Antibiotics*.

[2] Porreca A. M., Sullivan K. V., Gallagher J. C. (2018). The epidemiology, evolution, and treatment of KPC-producing organisms. *Current Infectious Disease Reports*.

[3] Satlin M. J., Chen L., Patel G. (2017). Multicenter clinical and molecular epidemiological analysis of bacteremia due to carbapenem-resistant enterobacteriaceae (CRE) in the CRE epicenter of the United States. *Antimicrobial Agents and Chemotherapy*.

[4] Falagas M. E., Tansarli G. S., Karageorgopoulos D. E., Vardakas K. Z. (2014). Deaths attributable to carbapenem-resistant Enterobacteriaceae infections. *Emerging Infectious Diseases*.

[5] Huang W., Qiao F., Zhang Y. (2018). In-hospital medical costs of infections caused by carbapenem-resistant *Klebsiella pneumoniae*. *Clinical Infectious Diseases*.

[6] French C. E., Coope C., Conway L. (2017). Control of carbapenemase-producing Enterobacteriaceae outbreaks in acute settings: an evidence review. *Journal of Hospital Infection*.

[7] Jayol A., Poirel L., Dortet L., Nordmann P. (2016). National survey of colistin resistance among carbapenemase-producing Enterobacteriaceae and outbreak caused by colistin-resistant OXA-48-producing *Klebsiella pneumoniae*, France, 2014. *Euro Surveillance*.

[8] Thoma R., Seneghini M., Seiffert S. N. (2022). The challenge of preventing and containing outbreaks of multidrug-resistant organisms and Candida auris during the coronavirus disease 2019 pandemic: report of a carbapenem-resistant Acinetobacter baumannii outbreak and a systematic review of the literature. *Antimicrobial Resistance and Infection Control*.

[9] Sun Jin L., Fisher D. (2021). MDRO transmission in acute hospitals during the COVID-19 pandemic. *Current Opinion in Infectious Diseases*.

[10] Ergen P., Kocoglu M. E., Nural M. (2022). Carbapenem-resistant *Klebsiella pneumoniae* outbreak in a COVID-19 intensive care unit; a case-control study. *Journal of Chemotherapy*.

[11] Gregory C. J., Llata E., Stine N. (2010). Outbreak of carbapenem-resistant *Klebsiella pneumoniae* in Puerto Rico associated with a novel carbapenemase variant. *Infection Control & Hospital Epidemiology*.

[12] Epstein L., Hunter J. C., Arwady M. A. (2014). New Delhi metallo-beta-lactamase-producing carbapenem-resistant *Escherichia coli* associated with exposure to duodenoscopes. *JAMA*.

[13] Di Tella D., Tamburro M., Guerrizio G., Fanelli I., Sammarco M. L., Ripabelli G. (2019). Molecular Epidemiological Insights into Colistin-Resistant and Carbapenemases-Producing Clinical *Klebsiella pneumoniae* Isolates. *Infection and Drug Resistance*.

[14] World Health Organisation (2017). *Guidelines for the Prevention and Control of Carbapenem-Resistant Enterobacteriaceae, Acinetobacter Baumannii and Pseudomonas aeruginosa in Health Care Facilities*.

[15] Feng C., Li J., Sun W., Zhang Y., Wang Q. (2016). Impact of ambient fine particulate matter (PM2.5) exposure on the risk of influenza-like-illness: a time-series analysis in Beijing, China. *Environmental Health*.

[16] Song X., Xiao J., Deng J., Kang Q., Zhang Y., Xu J. (2016). Time series analysis of influenza incidence in Chinese provinces from 2004 to 2011. *Medicine*.

[17] Balinskaite V., Johnson A. P., Holmes A., Aylin P. (2019). The impact of a national antimicrobial stewardship program on antibiotic prescribing in primary care: an interrupted time series analysis. *Clinical Infectious Diseases*.

[18] Sax H., Allegranzi B., Uckay I., Larson E., Boyce J., Pittet D. (2007). “My five moments for hand hygiene”: A user-centred design approach to understand, train, monitor and report hand hygiene. *Journal of Hospital Infection*.

[19] Tomczyk S., Zanichelli V., Grayson M. L. (2019). Control of carbapenem-resistant enterobacteriaceae, acinetobacter baumannii, and *Pseudomonas aeruginosa* in healthcare facilities: A systematic review and reanalysis of quasi-experimental studies. *Clinical Infectious Diseases*.

[20] World Health Organisation (2009). *WHO Guidelines on Hand Hygiene in Health Care: First Global Patient Safety Challenge Clean Care Is Safer Care*.

[21] Gu D., Dong N., Zheng Z. (2018). A fatal outbreak of ST11 carbapenem-resistant hypervirulent *Klebsiella pneumoniae* in a Chinese hospital: a molecular epidemiological study. *The Lancet Infectious Diseases*.

[22] Schwaber M. J., Lev B., Israeli A. (2011). Containment of a country-wide outbreak of carbapenem-resistant *Klebsiella pneumoniae* in Israeli hospitals via a nationally implemented intervention. *Clinical Infectious Diseases*.

[23] Abboud C. S., de Souza E. E., Zandonadi E. C. (2016). Carbapenem-resistant Enterobacteriaceae on a cardiac surgery intensive care unit: successful measures for infection control. *Journal of Hospital Infection*.

[24] van Loon K., Voor In ‘t Holt A. F., Vos M. C. (2018). A systematic review and meta-analyses of the clinical epidemiology of carbapenem-resistant enterobacteriaceae. *Antimicrobial Agents and Chemotherapy*.

[25] Daikos G. L., Tsaousi S., Tzouvelekis L. S. (2014). Carbapenemase-producing *Klebsiella pneumoniae* bloodstream infections: lowering mortality by antibiotic combination schemes and the role of carbapenems. *Antimicrobial Agents and Chemotherapy*.

[26] Chea N., Bulens S. N., Kongphet-Tran T. (2015). Improved phenotype-based definition for identifying carbapenemase producers among carbapenem-resistant enterobacteriaceae. *Emerging Infectious Diseases*.

[27] Hayden M. K., Lin M. Y., Lolans K. (2015). Prevention of colonization and infection by *Klebsiella pneumoniae* carbapenemase-producing enterobacteriaceae in long-term acute-care hospitals. *Clinical Infectious Diseases*.

